# Effects of Alloying Element Ca on the Corrosion Behavior and Bioactivity of Anodic Films Formed on AM60 Mg Alloys

**DOI:** 10.3390/ma10010011

**Published:** 2016-12-26

**Authors:** Anawati Anawati, Hidetaka Asoh, Sachiko Ono

**Affiliations:** 1 Research Institute for Science and Technology, Kogakuin University, 2665-1 Nakano, Hachioji, Tokyo 192-0015, Japan; anawati04@ui.ac.id (A.A.); asoh@cc.kogakuin.ac.jp (H.A.); 2 Department of Physics, Faculty of Mathematics and Natural Sciences, University of Indonesia, Depok 16424, Indonesia; 3 Department of Applied Chemistry, Kogakuin University, 2665-1 Nakano, Hachioji, Tokyo 192-0015, Japan

**Keywords:** magnesium alloy, anodic films, corrosion, polarization, biodegradable

## Abstract

Effects of alloying element Ca on the corrosion behavior and bioactivity of films formed by plasma electrolytic oxidation (PEO) on AM60 alloys were investigated. The corrosion behavior was studied by conducting electrochemical tests in 0.9% NaCl solution while the bioactivity was evaluated by soaking the specimens in simulated body fluid (SBF). Under identical anodization conditions, the PEO film thicknesses increased with increasing Ca content in the alloys, which enhanced the corrosion resistance in NaCl solution. Thicker apatite layers grew on the PEO films of Ca-containing alloys because Ca was incorporated into the PEO film and because Ca was present in the alloys. Improvement of corrosion resistance and bioactivity of the PEO-coated AM60 by alloying with Ca may be beneficial for biodegradable implant applications.

## 1. Introduction

Magnesium (Mg) and its alloys are suitable candidates for biodegradable implants because of their high strength-to-weight ratio, appropriate mechanical properties (e.g., Young’s modulus), and excellent biocompatibility in human body fluids [1,2,3]. However, Mg is electrochemically active in most applicable environments. In general, two methods are used to improve the corrosion resistance of Mg: an intrinsic approach involving the combination of a proper material processing method, the selection of appropriate alloying elements, and control of the microstructure; and an extrinsic approach involving surface treatments (e.g., anodization and coating) [4]. A combination of alloying and anodization techniques is often used to achieve a certain degree of corrosion protection.

Recently, researchers are investigating the addition of Ca as an alloying element to pure Mg [5,6,7,8] and commercial Mg alloys [9,10,11,12] to reduce the corrosion rate of biodegradable Mg as well as accelerate new bone formation. Ca is the main component of the human bone [1], and the release of Ca^2+^ ions can improve the bioactivity of an implant to quicken the bone healing process. The addition of Ca as an alloying element tends to refine the metal grains of Mg alloys [5,6,7,8] and reduce the number of intermetallic (Mg_17_Al_12_) grains formed [9,10,11,12], which improves the alloy’s corrosion resistance. For example, a reduction in the corrosion rate of AZ91D alloy measured in 5 wt % NaCl solution was obtained by alloying with approximately 2 wt % Ca; however, at Ca concentrations greater than 2 wt %, the corrosion rate began to increase gradually [9]. Alloying with Ca also represents an alternative method to reduce the Al content in commercial alloys while maintaining their corrosion resistance. Kannan and Raman [10] reported that AZ61 containing 0.4 wt % Ca alloy exhibited corrosion resistance similar to that of AZ91 alloy when tested in simulated body fluid (SBF).

Anodization or coating of Mg alloys’ surfaces is an effective approach to limit contact between the metal surface and the corrosive environment, thereby improving their corrosion resistance. Plasma electrolytic oxidation (PEO) is a technique commonly used to grow a ceramic type of oxide that substantially improves the corrosion and mechanical properties of Mg alloys [13,14,15]. PEO coating on Mg alloys remarkably improved the mechanical properties including wear resistance, surface hardness, and elastic modulus [16]. PEO films are more stable and inhibit corrosion better than chemical conversion layers [15,17]. Mg implant specimens coated with PEO film exhibited better long-term degradation performance in SBF than the non-coated specimen [18]. Furthermore, in vivo study of Mg-Zn-Ca alloys in a mouse model confirmed that PEO coating on the alloys decreased the corrosion rate in in vivo environment [19]. The properties and chemical composition of the PEO layer are determined by the alloying elements, bath solution, and processing parameters [20]. In this study, we aim to clarify the effects of alloying element Ca on the thickness and composition of the PEO film formed on AM60 Mg alloys, where the PEO film further affects the corrosion resistance and bioactivity of the specimens. Most previous studies [5,6,7,8,9,10,11,12] investigated only the effect of Ca on the corrosion behavior of uncoated Mg alloys. However, in the present study, combined effects of alloying element Ca and PEO coating on the corrosion behavior of AM60 specimens were studied using polarization tests in physiological 0.9% NaCl solution; in addition, the bioactivity of the coated specimens was investigated by immersion tests in SBF.

## 2. Results

### 2.1. Substrate Microstructure and Composition

**Figure 1:**
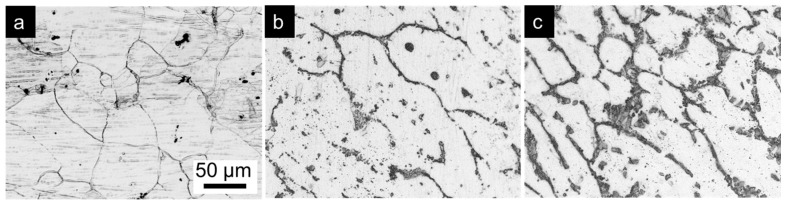
Optical microscopy images of (**a**) AM60; (**b**) AM60-1Ca; and (**c**) AM60-2Ca substrates showing the effect of Ca on the microstructure. The scale bar in image (**a**) applies to all images.

Figure 1 shows the microstructure of AM60 specimens containing different amounts of Ca. The AM60, AM60-1Ca, and AM60-2Ca are designated for base AM60, AM60 containing 1 wt % Ca, and AM60 containing 2 wt % Ca, respectively. All of the specimens exhibited nearly equiaxed grains with variation in size between 10 and 100 µm. Alloying with Ca slightly refined the grain size of the AM60 alloy. The surface of the AM60 specimen shown in Figure 1a was decorated by a few intermetallic particles with a diameter of ~1 µm; these particles appeared as black spots located mainly at grain boundaries. The number and size of the intermetallic particles were enhanced substantially by the addition of 1 and 2 wt % Ca to the alloys (Figure 1b,c). A reticular distribution of the intermetallic phase along the grain boundaries, forming a network-like structure, was observed in both of the Ca-containing specimens; by contrast, mainly discrete particles were observed in the grain interior. Most of the discrete particles in the AM60-1Ca specimen were larger (i.e., in the range from 2 to 10 µm) than those observed in the base alloy. An increase in the Ca content in the alloy to 2 wt % thickened the intermetallic phase along the grain boundaries and resulted in no discrete particles in the grain bodies.

**Figure 2:**
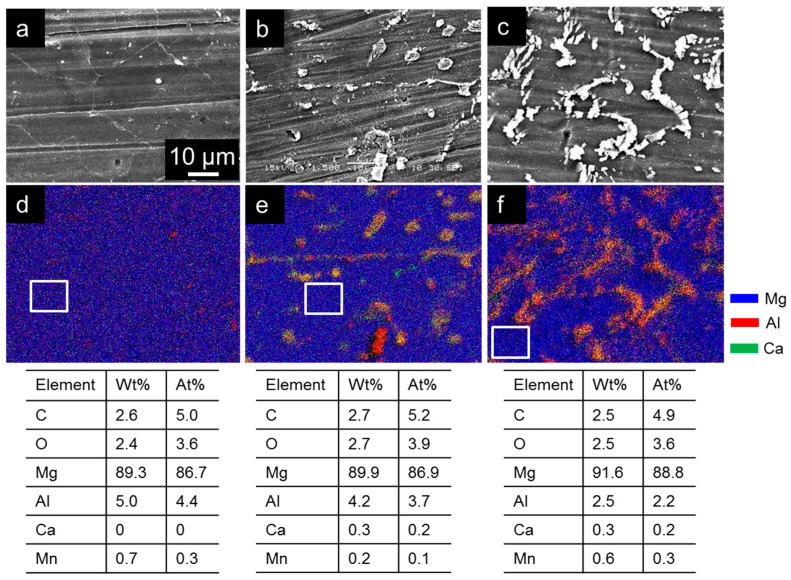
Plane-view scanning electron microscopy (SEM) images of (**a**) AM60; (**b**) AM60-1Ca; and (**c**) AM60-2Ca substrates and the corresponding energy-dispersive X-ray spectroscopy (EDX) maps (**d**–**f**) showing the distribution of Mg (blue), Al (red), and Ca (green); the tables show the elemental compositional inside the square drawn in each image. The scale bar in image (**a**) applies to all images.

**Figure 3:**
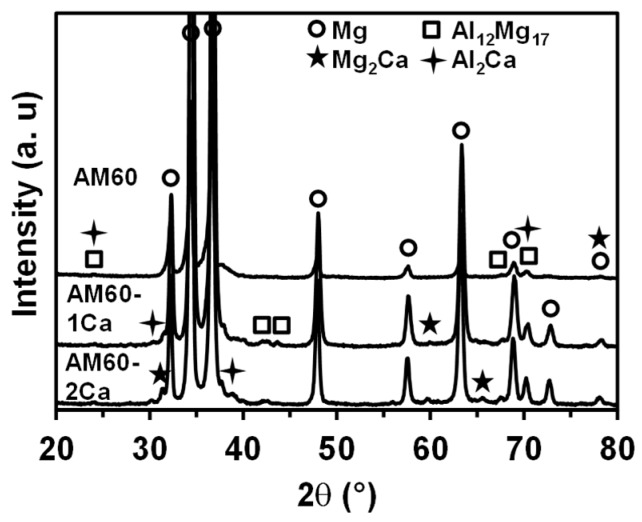
X-ray diffraction (XRD) patterns of the AM60, AM60-1Ca, and AM60-2Ca substrates.

Figure 2 shows the energy-dispersive X-ray spectroscopy (EDX) maps for Mg (blue), Al (red), and Ca (green) taken from the surfaces of AM60, AM60-1Ca, and AM60-2Ca; the tables included in this figure show the metal-matrix composition in the area inside the square in each image. The AM60 specimen exhibited a relatively uniform distribution of Al in the matrix, giving a slight purple color in the map with traces amount of the intermetallic phase (Figure 2a). X-ray diffraction (XRD) analysis (Figure 3) indicated that the main intermetallic phase was Al_12_Mg_17_. The intermetallic phase shown in both the AM60-1Ca and AM60-2Ca specimens gave strong intensities for a combination of Ca and Al, shown as yellow, and for a combination of Ca and Mg, shown as cyan, in the maps in Figure 2b,c. The addition of Ca to AM60 alloys induced precipitation of a substantial amount of Al–Ca phase and a small amount of Mg–Ca phase. The map of AM60-2Ca (Figure 2c) showed mainly a continuous distribution of the intermetallic phase along the grain boundaries; distinguishing individual phases along the line is difficult. EDX analysis of the metal-matrix areas inside the square area in Figure 2a–c detected a lower Al content in the AM60-1Ca and AM60-2Ca alloys compared to that in the base alloy. The Al content in the matrix decreased from 5.0 to 2.5 wt % with increasing amount of Ca added to the alloys. The Ca concentration in AM60-1Ca and AM60-2Ca metal matrices was similar at 0.3 wt %, which is quite low relative to the total concentration of 1–2 wt %, suggesting low solubility of Ca in Mg.

The XRD patterns of the three substrates are presented in Figure 3. The base alloy was composed of a primary Mg phase and a secondary phase, Al_12_Mg_17_. The Al_12_Mg_17_ peaks at high angles of 67.8°, 70.4°, and 72.8° increased in intensity with increasing Ca content in the alloys, indicating an increase in the intermetallic content. The presence of new phases, Al_2_Ca and Mg_2_Ca, in the Ca-containing alloys was confirmed by the appearance of new peaks at 59.9° and 65.7°, which correspond to the Al_2_Ca phase, and at 30.1° and 39.3°, which correspond to the Mg_2_Ca phase.

### 2.2. Formation of PEO Films

**Figure 4:**
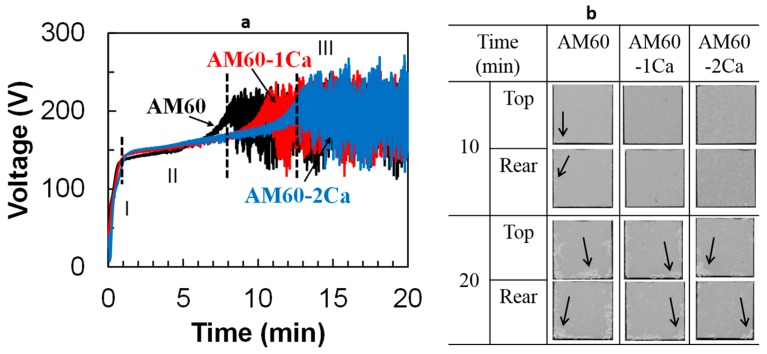
(**a**) Voltage–time curves during anodization of AM60, AM60-1Ca, and AM60-2Ca specimens in 0.5 mol·dm^−3^ Na_3_PO_4_ solution at 25 °C; and (**b**) the appearance of the specimens after anodization. The areas affected by strong discharge are marked by arrows in image (**b**).

The PEO film was formed by anodization of the AM60 specimens in 0.5 mol·dm^−3^ Na_3_PO_4_ solution at 25 °C. Figure 4 shows the voltage–time curves for the three alloys during anodization in 0.5 mol·dm^−3^ Na_3_PO_4_ solution; it also shows the appearance of the specimens after 10 and 20 min of anodization. The curves can be divided into three stages: stage I, linear growth (*V* < 140 V); stage II, uniform sparking (140 V < *V* < *V*_critical_); and stage III, strong and localized sparking (*V* > *V*_critical_). Stage I is indicated by an initial rapid increase in potential, where a relatively thin barrier oxide layer was formed. Stage II started as the thin-layer breakdown, and fine white sparking appeared uniformly on the specimen surfaces, which contributed to uniform film thickening. The voltage then increased slowly with time to a critical voltage of approximately 200 V, where intense sparking discharges began to occur at local defects, indicating the onset of stage III. The strong discharge caused large oscillation in the voltage between 150 and 250 V. The critical voltage was achieved at 8 min for the base alloy and was delayed to 10 and 12 min for AM60-1Ca and AM60-2Ca, respectively. Figure 4b shows that the PEO film resulting from 10 min anodization of AM60-1Ca and AM60-2Ca specimens exhibited a uniform film. Meanwhile, the film formed on AM60 showed a local thickening viewed as white areas, marked by arrows, near the specimen edges, as a result of strong discharge. The films formed after 10 min of anodization exhibited an average thickness of 22, 26, and 28 µm for the specimens containing 0, 1, and 2 wt % Ca, respectively, as measured using a coating thickness meter.

The effect of strong discharge was clarified at 20 min of anodization time. The white areas on the film surface expanded along the specimen edges (Figure 4b). The thickness of the white areas was approximately 50–80 µm. Excluding the white areas, the average film thicknesses after 20 min of anodization were 29, 33, and 34 µm for AM60, AM60-1Ca, and AM60-2Ca, respectively. Therefore, in the average area, the growth rate of the film during fine plasma discharge appears to be more than twice that obtained during strong plasma discharge. If the thicker oxide area (white areas) were included, the average thicknesses of the PEO film on AM60, AM60-1Ca, and AM60-2Ca would be 42, 38, and 35 µm, respectively. The primary focus in this study was the film formed after 20 min of anodization, where the maximum film thickness is obtained without dissolving the specimen edges.

### 2.3. Structure and Composition of PEO Films

**Figure 5:**
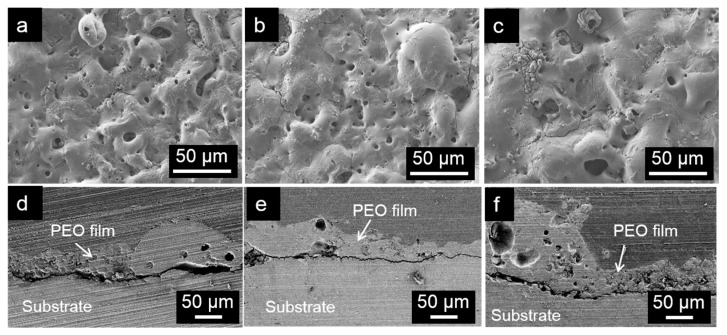
The film structure resulting from 20 min anodization in 0.5 mol·dm^−3^ Na_3_PO_4_ solution at 25 °C in plane and cross-sectional views on (**a**,**d**) AM60; (**b**,**e**) AM60-1Ca; and (**c**,**f**) AM60-2Ca.

There was no significant difference observed in the structure of the films formed on AM60, AM60-1Ca, and AM60-2Ca specimens, as shown in Figure 5. All of the films exhibited uneven structures and were decorated by spherical pores and cracks. Entrapment of evolved gas during anodization caused the formation of such pores, whereas cracks were formed as a result of the release of mechanical stress because of melting and rapid cooling of a region of the oxide film. The thickness of the oxide film fluctuated strongly, following a lava-like structure, as clearly shown in the cross-section images in Figure 5d–f. The pore size inside the thicker part of the film was larger compared to that of pores observed in the thinner part. EDX analysis of the film formed on the AM60 specimen indicated that Mg, P, and O were the main elements in the film, with concentrations of 17.5 at %, 12.9 at %, and 58.0 at %, respectively. The concentrations did not vary for the film formed on the Ca-containing alloys. Other elements detected in the oxide film were Na (~5.7 at %), Al (0.6 at %), and C (5.0 at %). The signal intensity for Ca approached the noise level, giving concentrations of approximately 0.1 and 0.3 at % in the films formed on the AM60-1Ca and AM60-2Ca specimens, respectively.

**Figure 6:**
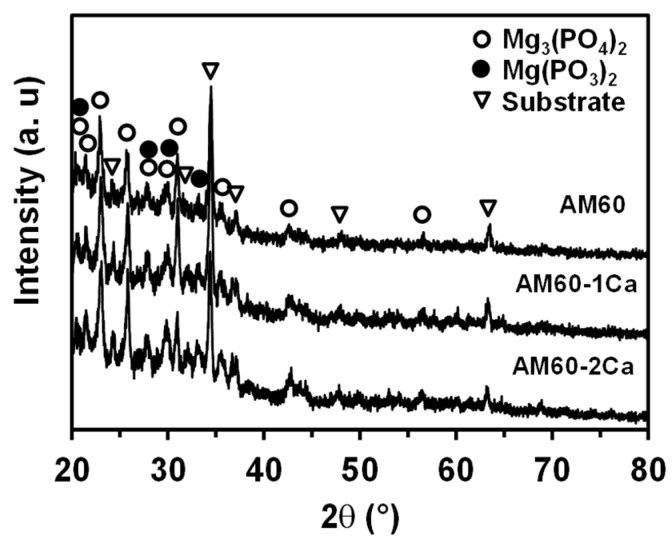
XRD patterns of the plasma electrolytic oxidation (PEO) film formed AM60, AM60-1Ca, and AM60-2Ca specimens for 20 min anodization in 0.5 mol·dm^−3^ Na_3_PO_4_ solution at 25 °C.

The heat produced by plasma discharge during anodization yielded the formation of both amorphous and crystalline oxides. The XRD patterns of the PEO films formed on the three alloys are presented in Figure 6. A noticeable broad peak was present between 15° and 40°; this peak is attributed to an amorphous state, whereas the serial tiny peaks that decorated the broad peak are attributed to the presence of a microcrystalline phase. The peaks that appeared in the three diffraction patterns were located at the same 2θ positions, implying similar film compositions. The amorphous phase might consist of MgO, Mg(OH)_2_, Mg_3_(PO_4_)_2_, and Mg(PO_3_)_2_ phases. The crystalline phases of the oxide were Mg(PO_3_)_2_ and Mg_3_(PO_4_)_2_. The mechanism for the formation of the oxide phases is discussed in our previous research [21]. XRD analysis did not indicate the presence of a Ca compound in the PEO film.

**Figure 7:**
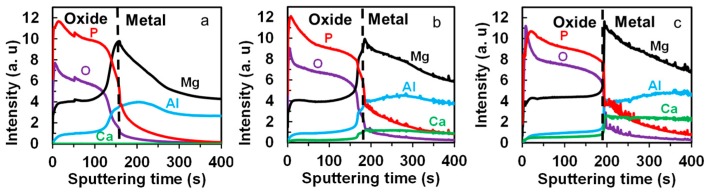
Glow-discharge optical emission spectroscopy (GDOES) elemental depth profiles of PEO films formed on (**a**) AM60; (**b**) AM60-1Ca; and (**c**) AM60-2Ca specimens after 20 min of anodization in 0.5 mol·dm^−3^ Na_3_PO_4_ solution at 25 °C.

Depth-profile glow-discharge optical emission spectroscopy (GDOES) analysis was performed on the specimens anodized for 20 min, with the main purpose of determining if Ca was present in the PEO film. The results are shown in Figure 7. The dashed line denotes the oxide–film interface, as judged on the basis of the decrease in O and P profiles approaching zero, and an increase in the Al and Ca profiles approaching the stable bulk intensity. The oxide–metal interface shifted towards longer sputtering time from 160 s to 190 s with increasing Ca content in the alloys indicating thicker film in specimens containing a higher Ca percentage. The analyzed area was at the specimen center, which did not include the thick film areas (white areas denoted by arrows in Figure 4b). There is a high risk of gas leakage during GDOES measurements if the specimen surface with large roughness is exposed to the O-ring that seals the target area. The Ca profile for the anodized AM60 specimen showed a constant intensity of zero throughout the time scale because neither the film nor the metal contained Ca. The intensity level of Ca in both the film and bulk metal regions increased with increasing Ca content in the alloys, suggesting that the oxide film formed on AM60-1Ca and AM60-2Ca specimens contained Ca, and that the concentration of Ca in the oxide film increased with increasing Ca concentration in the bulk metal. The depth profiles also confirmed that the main constituent elements of the PEO film were P, O, and Mg. Al was present in the films at concentrations less than half of its bulk concentration in all three specimens. A broad peak appeared at the oxide–metal interface of Al and Mg curves was typically caused by interfacial roughness and by non-uniform oxide thickness.

**Figure 8:**
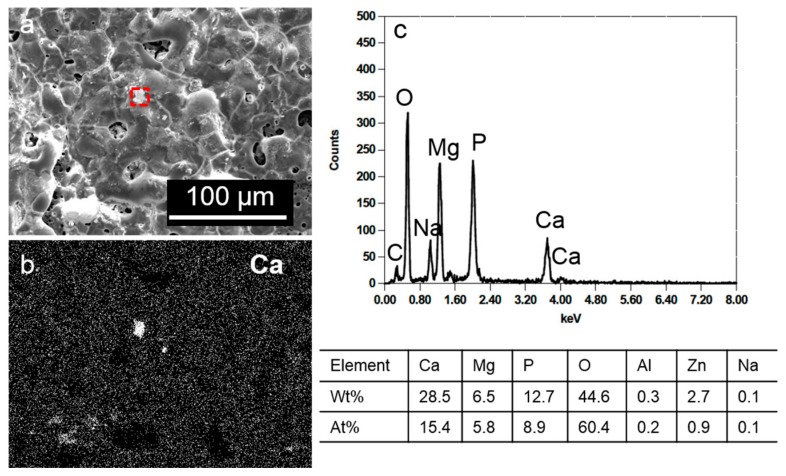
(**a**) SEM micrograph of the PEO film resulting from 10 min anodization of AM60-1Ca specimen in 0.5 mol·dm^−3^ Na_3_PO_4_ solution at 25 °C; (**b**) the corresponding EDX maps for Ca; and (**c**) the spectra from red square area in image (**a**) and the elemental composition.

To confirm the incorporation of Ca into the PEO film, we performed EDX analysis on the film formed at the shorter anodization time of 10 min. Figure 8 shows a scanning electron microscopy (SEM) micrograph of the PEO film formed after 10 min anodization on AM60-1Ca specimens and the corresponding EDX Ca maps. The Ca compound was detected randomly on the film, and its size varied. Most of the compound was smeared within the film, whereas the remainder was agglomerated into approximately 10 µm particles. The spectrum collected in an area around the Ca compound appeared at the center of Figure 8a (red-dashed box) is presented in Figure 8c, together with the elemental composition in the table below the spectrum. The compound mainly contained Ca, O, Mg, and P, and a portion of the O, Mg, and P signals stem from the PEO film. The compound might be composed of Ca_3_(PO_4_)_2_ or CaO. Because a low Ca content was observed in the film formed after 20 min of anodization, an extension of the anodization time to 20 min possibly dissolved part of the compound during strong discharge, whereas the rest of the compound might be covered by the newly grown film.

### 2.4. Electrochemical Corrosion

**Figure 9:**
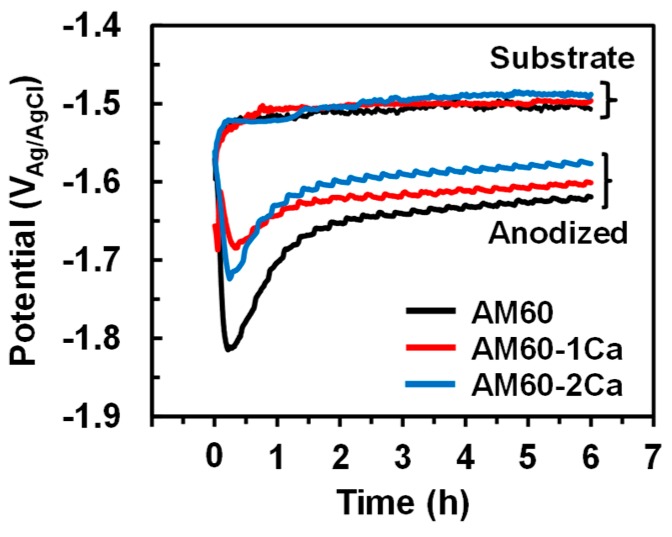
The open circuit potential (OCP) of substrates and anodized specimens of AM60, AM60-1Ca, and AM60-2Ca in 0.9% NaCl solution at 37 °C.

Figure 9 shows the temporal change of open circuit potential (OCP) or free potential of both the substrate and the anodized specimens of AM60, AM60-1Ca, and AM60-2Ca measured in 0.9% NaCl solution at 37 °C. The measurements were repeated at least three times for each specimen. The substrates demonstrated similar behavior of rapidly increasing potential to −1.49 V_Ag/AgCl_ after approximately 20 min. The potential remained at −1.49 V_Ag/AgCl_ for the AM60 substrate and increased slowly to −1.50 V_Ag/AgCl_ and −1.51 V_Ag/AgCl_ for the AM60-1Ca and AM60-2Ca substrates, respectively, after 6-h measurements. The curves of the anodized specimens were shifted to negative potentials relative to the curves of the substrates. The anodized specimens exhibited potential transients at the beginning of the measurements; these transients likely corresponded to the time required for the solution to infiltrate the film. The potentials of the anodized specimens modestly increased to −1.64, −1.62, and −1.60 V_Ag/AgCl_ for the AM60, AM60-1Ca, and AM60-2Ca specimens, respectively, after approximately 2 h of exposure.

**Figure 10:**
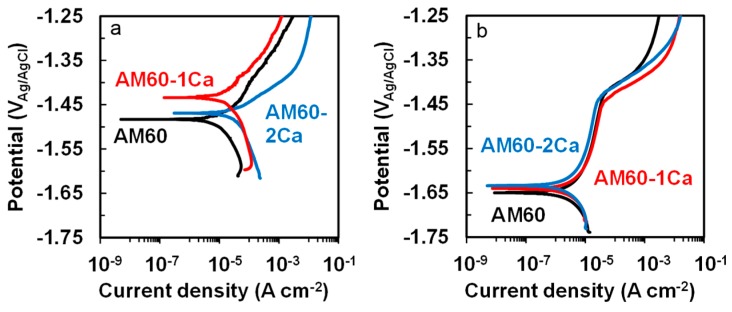
Potentiodynamic polarization curves of (**a**) substrates and (**b**) anodized specimens of AM60, AM60-1Ca, and AM60-2Ca in 0.9% NaCl solution at 37 °C.

Figure 10 shows the effect of Ca on the potentiodynamic polarization curves of the substrate and anodized AM60 specimens in 0.9 wt % NaCl solution at 37 °C. The polarization measurements were performed after the OCP was stabilized to obtain reproducible results. The substrates required 40 min to achieve a stable potential in the solution, whereas anodized specimens required 2 h, as also shown in Figure 9. Figure 10a shows that the cathodic current densities of the AM60-1Ca and AM60-2Ca substrates were higher than that of the AM60 substrate, which is attributed to the higher volume fraction of the intermetallic phase. The AM60 substrate exhibited a corrosion potential at −1.48 V_Ag/AgCl_; this potential was substantially increased to a nobler potential of −1.44 V_Ag/AgCl_ by the addition of 1 wt % Ca, but shifted back to −1.47 V_Ag/AgCl_ when the Ca content was increased to 2 wt %. This shift of corrosion potential resulted in an increase of the corrosion current density. The corrosion current density of the AM60 substrate increased slightly from 1.16 × 10^−5^ A·cm^−2^ to 1.44 × 10^−5^ A·cm^−2^ with the addition of 1 wt % Ca in the alloy, and further to 2.67 × 10^−5^ A·cm^−2^ in the 2 wt % Ca content case.

Figure 10b shows that the polarization curves of the anodized specimens shifted toward lower potentials relative to the curves of the substrates. However, the anodized specimens generated corrosion current densities an order of magnitude lower. The corrosion potentials of the three anodized specimens were −1.65, −1.64, and −1.63 V_Ag/AgCl_, and similar corrosion current densities of approximately 2.0 × 10^−6^ A·cm^−2^. The corrosion current densities of the anodized specimens were approximately an order of magnitude lower than those of the substrates. A wide passivation-like region existed between the corrosion and breakdown potentials of the anodized specimens. Along the passivation-like region, the anodic current density did not vary substantially with increasing potential until a breakdown potential, corresponded to pitting potential, commenced at −1.44 V_Ag/AgCl_, where the anodic current began to increase drastically.

### 2.5. In Vitro Bioactivity

**Figure 11:**
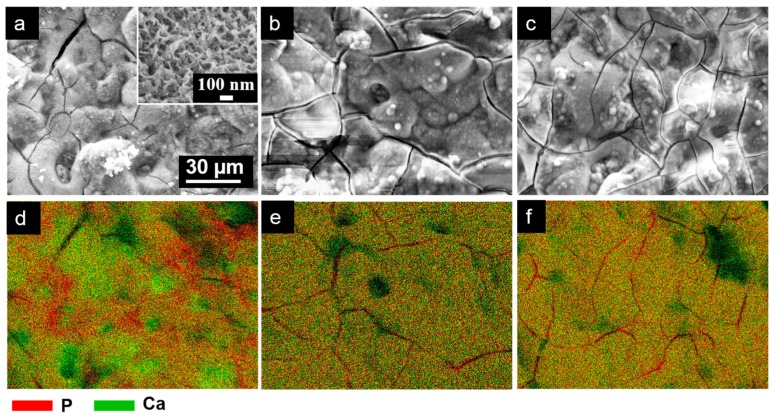
(**a**–**c**) Surface morphology and (**d**–**f**) EDX spectra for P and Ca of the apatite layer formed on the anodized surfaces of AM60, AM60-1Ca, and AM60-2Ca, respectively, after immersion in SBF for 14 days.

**Figure 12:**
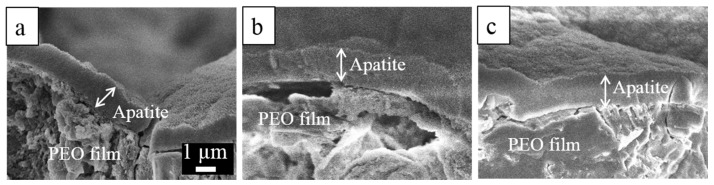
Cross-sectional SEM images of the apatite layer formed on the anodized surfaces of (**a**) AM60; (**b**) AM60-1Ca; and (**c**) AM60-2Ca after incubation in SBF for 14 days.

The metal substrates and the anodized specimens were subjected to in vitro tests by incubating the specimens in SBF at 37 °C for 14 days. All of the anodized surfaces were covered by a thick deposit layer after 14 days of immersion in SBF, as shown in the SEM micrograph in Figure 11a–c. The deposit layers formed on the three anodized specimens exhibited a spongy structure, which is typical of the apatite layer formed in SBF, as shown in a higher-magnification image in the inset Figure 11a. The cracks that appeared in the layer occurred during drying and further dehydration under beam exposure. The cross-sectional SEM images shown in Figure 12 revealed that the thicknesses of the apatite layers grown on the three anodized specimens were approximately similar, in the range of 1–2 µm. However, the EDX maps in Figure 11d–f indicate that thicker apatite layers were formed on the PEO films of Ca-containing alloys considering the variation in proportion of the P and Ca intensities and the fact that a similar form of apatite, hydroxyapatite (HA), and Ca_3_PO_4_ were detected by XRD analysis (not shown) of the three specimens. The maps in Figure 11 show the distribution of elements P (red) and Ca (green). The surface maps of as-anodized specimens typically gave a strong red color because of a strong signal from P in the PEO film. The apatite composed of both P and Ca, and therefore its existence, is shown as a yellow-greenish color as a combination of red and green colors. After immersion in SBF, part of the signal from the PEO film appeared slightly reddish in the map for the AM60 specimen in Figure 11d, suggesting that a thin apatite layer had covered the PEO film. Meanwhile, the other areas covered by a thick apatite layer gave a yellow-greenish color. The map for AM60-1Ca and AM60-2Ca in Figure 11e,f shows uniformly yellow-to-greenish color, indicating that a thick apatite layer distributed evenly on the PEO film. The apatite layer is composed of Ca, P, Mg, and O. The elements Mg and P stemming from the underlying PEO film were still detected. The layer grown on the PEO film of the AM60 specimen exhibited a Ca/P atomic ratio of 0.77. The highest Ca/P ratio of 1.25 was obtained for the deposit layer formed on AM60-1Ca, whereas the layer formed on the anodized AM60-2Ca specimen exhibited a Ca/P ratio of 1.17.

## 3. Discussion

Surface investigation on PEO films of AM60 magnesium alloys revealed that the thickness and composition of the PEO films varied with the microstructure and composition of the substrates. The addition of 1–2 wt % Ca modified the microstructure of the AM60 alloys by refining the metal grains and inducing the formation of a greater amount of intermetallic phase. The presence of Ca enhanced the precipitation of the Al_12_Mg_17_ phase in addition to the formation of new phases Al_2_Ca and Mg_2_Ca, as indicated by the EDX maps (Figure 2) and XRD analysis results (Figure 3). This result is contrary to the previously reported behavior for AZ91, where the addition of Ca reduced the amount of Al_12_Mg_17_ intermetallic in the alloys [9,10,11]. The reason for such contradictory behavior is unclear. The results of the present study suggest that the presence of Ca dissolved in the solid solution matrix might reduce Al–Mg bonding in the matrix of AM60 alloys. Thermodynamically, the maximum solubility of Ca in the Mg lattice was ~0.8 wt % at 516.5 °C and the solubility decreased with decreasing temperature [22]. The metal matrix of AM60-1Ca and AM60-2Ca contained only ~0.3 wt % Ca; the rest of the Ca was present as intermetallic phases. The increased amount of intermetallic phase in the presence of Ca reduced the Al content in the surrounding metal matrix of AM60 (Figure 2). Al was consumed for the formation of high fractions of Al_2_Ca and Al_12_Mg_17_ intermetallics.

The depletion of the Al content in the matrix and the existence of the Al_2_Ca phase resulted in a delay time for the formation of strong plasma discharge during PEO. The intense plasma discharge was formed only after the film attained high resistivity. The incorporation of aluminum into the PEO film contributed to an enhancement of the film passivity/resistivity. The time required to reach the critical voltage for intense plasma decreased linearly with increasing Al content in the Mg alloys, i.e., Mg alloy containing 3 wt % Al (AZ31) exhibited a critical voltage after 16 min [21], which was twice the time required for AM60 alloy under identical anodization conditions. The effect of Al on the anodization process of AZ alloys is under investigation and will be published in a separate paper. The results showed that the addition of each 1 wt % Al decreased the critical voltage time by approximately 2 min. Zn played a similar role in decreasing the time for critical voltage by 2 min for every 1 wt % Zn. The decrease of Al concentration in the metal matrix of AM60 alloys with increasing Ca content delays the formation of high resistivity in the film and, thus, the occurrence of strong discharge. The presence of intermetallic Al_2_Ca in AM60 alloys containing Ca can also retard the onset of intense plasma. The Al_2_Ca phase has a high melting point of 1097 °C [23] and is known to improve the heat resistance of Mg alloys [24].

Extension of the lifetime for fine plasma discharge with increasing Ca content would result in the formation of a thicker film on the Ca-containing alloys. The contribution of uniform film thickening to the corrosion resistance was greater than that of the local thickening. However, thickening by fine plasma is often not sufficient to protect the substrate surface from a corrosive environment. The increase in the degree of protection by the formation of a thicker oxide film resulting from strong plasma discharge was somewhat counterbalanced by an increase in crack density in the film. Full growth of the thicker film in a uniform manner therefore required a certain extension of the anodization time. A longer anodization time led to substantial thickening of the surface by strong plasma discharge, although the growth rate of the PEO film during strong plasma discharge was less than half that obtained during fine plasma discharge. A thicker oxide more effectively reduced the cathodic activity, i.e., hydrogen evolution, on the metal surface during the polarization measurements. Inhibition of the hydrogen evolution rate on the surface helped depress the corrosion potential of the anodized specimens relative to that of the substrate. The base alloy, which experienced a longer strong plasma discharge than the Ca-containing specimens, exhibited the lowest free corrosion potential (Figure 9). The depression of corrosion potential is typically observed for Mg alloys anodized in Na_3_PO_4_ solution [13]. The free corrosion potential of the anodized AM60 specimens became nobler by 20 mV for each 1 wt % Ca in the alloys. The polarization curves also showed a slight increase in corrosion potential of the anodized AM60 with increasing Ca content in the alloys (Figure 10b). The surfaces of anodized specimens were soon passivated as departed from the corrosion potential, as indicated by the formation of a passivation region between the corrosion and breakdown potentials in the polarization curves in Figure 10b. The relatively small anodic current generation was very likely due to the formation of a partially stable Mg(OH)_2_ layer inside the defects. At defects, such as pores and cracks in the PEO film, the solution was relatively stagnant since they did not allow free solution exchange with the bulk solution. The occluded area and pit tend to stabilize and stop the propagation of corrosion on Mg alloys [25]. The breakdown in passivity began to commence again at a potential of approximately −1.45 V_Ag/AgCl_, which was approximately the corrosion potential of the substrates (Figure 10a). At this potential, part of the areas at the interface between the film and metal substrate was possibly filled with the solution; thus, corrosion developed in a manner similar to the direct exposure of fresh AM60 substrates to the solution.

Under potentiodynamic polarization, the corrosion behavior of the bare AM60 substrates was sensitive to the microstructure. The network of intermetallic phase along the grain boundaries in the Ca-containing alloys ennobled the grain boundaries relative to the neighboring matrix. The nobler grain boundaries were beneficial to stop propagation of corrosion across the grain, as has been previously suggested [26]. The potentiodynamic polarization curves (Figure 10a) demonstrated that the corrosion potential of AM60 substrate containing 1 wt % Ca was significantly nobler than that of the base alloy. However, the addition of 2 wt % Ca did not shift the corrosion potential any further toward the positive direction relative to that of the AM60-1Ca substrate but instead decreased the corrosion potential toward that of the base alloy. The reduction of Al in the matrix and an increase in the amount of Mg_2_Ca phase in the AM60-2Ca alloy counterbalanced the improved corrosion resistance caused by the network barrier. The corrosion potential of Mg is known to decrease with decreasing Al content in Mg alloys [27]. The Mg_2_Ca phase has been reported to be electrochemically more active than Mg and assumes to role as an anode, unlike other intermetallic phases that are cathodes in relation to Mg [6].

The free corrosion potential of the bare AM60 substrates in NaCl solution was not sensitive to the variation of Ca content in the alloys. The corrosion potential decreased only slightly, from −1.49 to −1.51 V_Ag/AgCl_, by the addition of 1–2 wt % Ca to the alloys. The measured free corrosion potential is more representative of the corrosion of the solid solution matrix than of the intermetallics; therefore, the potential differences of the three AM60 substrates were relatively small. The protectiveness of the air-formed oxide layer on the AM60 substrate, which immediately increased in thickness during immersion in the solution, was not substantially affected by the presence of 1–2 wt % Ca in the alloys. The degree of protection of the native oxide was mainly influenced by the total concentration of alloying element Al. Nordlien et al. [28] have reported that the Al concentration in the natural oxide film reaches a saturation level of approximately 35 at %, providing optimum corrosion protection properties, when the Al content of the Mg alloy is equal to or greater than 4 wt %. The free corrosion potential curves of the anodized specimens gave more noticeable shift in the corrosion potential ~20 mV with increasing Ca concentration in the alloys. Confirming the results, slight ennoblement of about 10 mV was also observed in the polarization curves of the anodized specimens for every 1 wt % Ca addition in the alloys, indicating that the ennoblement of the corrosion potentials was attributed to the improvement in the protectiveness of PEO film. The PEO film gave a higher degree of protection to corrosion with increasing film thickness.

The corrosion potentials of the anodized specimens were approximately 200 mV lower than that of the unanodized specimens. The phenomenon is often reported [29] for PEO-coated Mg alloys, although improvement of corrosion resistance was observed. The shift in the corrosion potential towards negative direction relative to the substrate was very likely due to inhibition effect of the PEO layer for hydrogen evolution reaction on the surface, which suppressed the cathodic current, as indicated by the reduction of cathodic current about an order of magnitude in the polarization curves of the anodized specimens (Figure 10b). The PEO layer became an effective barrier that protects the substrate from corrosive solution as demonstrated by relatively constant anodic-current output of the anodized specimens in the order of 10 µA·cm^−2^ with increasing potential up to pitting potential at −1.44 V_Ag/AgCl_. At the pitting potential, pitting began to form at the defects in the PEO layer, such as cracks. Pitting occurred at the interface between substrate and the PEO layer that further led to local detachment of the PEO layer around the pit. The PEO-coated specimens are more prone to a pitting type of attack than those of the uncoated ones. The long-term degradation and pitting susceptibility of the PEO-coated AM60 alloys remained to be investigated. It will be interesting to test the effects of degradation on mechanical integrity since it was reported that [30] an enhancement in mechanical strength (20%) of Mg alloys is often obtained by coating with PEO.

Alloying element Ca is expected to improve not only the corrosion resistance, but also the bioactivity of the PEO film formed on AM60 specimens. The results showed that the apatite layers grown on the PEO films of AM60-1Ca and AM60-2Ca specimens during immersion in SBF were thicker, indicating greater bioactivity than that of the PEO film on AM60. The presence of Ca in the substrates and the incorporation of its compound in the PEO film accelerated the growth of apatite in SBF. During exposure in SBF, thinning of the PEO film led to an increase in surface roughness, which is favorable for the deposition of apatite on the surface, as previously reported [21]. The Ca compound in the film can become a cluster for apatite nucleation, whereas part of the compound that dissolved during film thinning may contribute to an increase in the degree of saturation of the SBF relative to apatite [14]. Ca from the underlying substrate was also released to the solution during corrosion at defects in the PEO film. The apatite growth rate on the PEO film surfaces of AM60 alloys containing Ca was therefore higher than that on the PEO film surfaces of the base alloys. A uniformly grown apatite layer should act as a barrier layer against corrosion and reduce the corrosion rate of the substrate during long-term exposure in a corrosive environment, as in the case of AZ31 alloys reported by us elsewhere [21].

Thicker apatite-layer growth on the anodized specimens of alloys containing Ca contributed to the increase in the Ca/P ratio. The Ca/P ratio of the apatite layers observed in the present cases was lower than the ratio for stoichiometric hydroxyapatite (HA, 1.67), mainly because the signal from the underlying PEO film, which contained a high concentration of P, contributed to the excess P concentration. The anodized specimens of AM60 alloys containing Ca show promise for biodegradable materials. Further research to evaluate cell response on the surface of anodized AM60 alloys containing Ca in cell culture medium will be motivating.

## 4. Materials and Methods

### 4.1. Specimen Preparation

The specimens used were rolled-plate commercial AM60 alloys with Ca contents of 0, 1, and 2 wt %. The alloying composition of the base AM60 alloy is listed in Table 1. The plates were cut into pieces to give a working area of 1.5 cm × 1.5 cm × 0.1 cm. For microstructure observations and corrosion tests, the specimens were ground to 1200-grit silicon carbide paper and then degreased in acetone in an ultrasonic bath for 3 min. To reveal the intermetallic phase, each specimen was etched in 4% HNO_3_ in ethanol. The microstructure study was performed using an Olympus BX51M optical microscope (Olympus Corporation, Tokyo, Japan).

### 4.2. Anodization

Before anodization, the as-received specimens were pretreated in a mixed acid solution of 8 vol % HNO_3_–1 vol % H_3_PO_4_ for 20 s and then washed with deionized (DI) water before being subsequently dipped in 5 wt % NaOH solution at 80 °C for 1 min. Each specimen was then washed again in DI water. Anodization was performed in 0.5 mol·dm^−3^ Na_3_PO_4_ solution at a constant current of 200 A·m^−2^ at 25 °C for 20 min. The parameters and conditions during anodization were similar to those previously reported [21]. The resulting oxide film thickness was measured using a Sanko dual-type (SME-1) coating thickness meter (Sanko Electronic Laboratory Co., Ltd., Kanagawa, Japan). The average thickness was calculated from the data obtained at 10 points measurements on each surface and rear surface.

### 4.3. Surface Analyses

Surface characterization of both anodized and unanodized specimens was conducted using field-emission scanning electron microscopy (FE-SEM; JEOL JSM-6701, JEOL Ltd., Tokyo, Japan). A thin Pt-Pd film was deposited onto the specimens’ surfaces prior to the SEM observations to minimize the charging effect. The elemental composition was analyzed using energy-dispersive X-ray spectroscopy (EDX; JEOL EX-54175JMU, JEOL Ltd., Tokyo, Japan); the EDX apparatus was attached to a scanning electron microscope (JEOL JSM-6380LA, JEOL Ltd., Tokyo, Japan). The chemical composition and crystalline state were analyzed by X-ray diffraction (XRD) analysis (Rigaku Rint 2000, Rigaku Corporation, Tokyo, Japan) at an incident angle of 1° using an accelerating voltage and current of 40 kV and 40 mA, respectively. Glow-discharge optical emission spectroscopy (GDOES; Jobin-Yvon JY5000RF, HORIBA, Ltd., Kyoto, Japan) was used to measure the elemental depth profile of the PEO film on a circular area with a diameter of 4 mm by Ar^+^-ion sputtering at 40 W.

### 4.4. Electrochemical Tests

The corrosion behavior of the specimens was elucidated by electrochemical tests using a physiological solution (0.9 wt % NaCl solution) at 37 °C; these tests were based on an ASTM G5 [31]. One surface of each specimen was exposed to the solution. The electrochemical tests were performed using an IviumStat potentiostat. Pt wire was used as the counter electrode, and an Ag/AgCl electrode was used as the reference electrode. The electrochemical cell conditions and arrangement are similar to those used in our previous research [21,32]. Potentiodynamic polarization tests were conducted at a sweep rate 1 mV·s^−1^ from 100 mV below the OCP and were terminated when the current output reached 30 mA. The corrosion potential and current density were estimated by Tafel extrapolation. Before the polarization tests, each specimen was maintained at the OCP until the potential stabilized.

### 4.5. In Vitro Immersion Tests

The in vitro corrosion tests were performed by incubating specimens individually in SBF at 37 °C. SBF10 with the ionic concentration shown in Table 2 was prepared as previously reported [33]. The solution pH was adjusted to 7.4 at 37 °C. The specimens, with a surface-to-volume ratio of 20 mL·cm^−2^, were exposed to SBF for 14 days. The solution was replaced every three days.

## 5. Conclusions

The effect of Ca on the corrosion behavior and bioactivity of PEO films formed on AM60 alloys containing 0, 1, and 2 wt % Ca was investigated; our conclusions are as follows:

1. The addition of Ca to the alloys slightly increased the PEO film thickness formed on AM60 alloys when constant-current anodization was performed.
2. Increasing Ca content in the alloys extended the lifetime of fine plasma discharge during PEO because of the depletion of Al in the metal matrix and the reduction of Mg–Al precipitate, which resulted in thicker PEO films.
3. The free corrosion potentials of the anodized AM60 specimens measured in 0.9% NaCl solution indicated slight ennoblement of the potential with increasing Ca concentration in the alloys. Similarly, the polarization curves for the anodized specimens shifted slightly to the nobler direction with increasing Ca content in the alloys. The improvement of corrosion resistance of the anodized AM60 specimens with increasing Ca content in the alloys was presumably attributable to the increase in PEO film thickness with increasing Ca concentration in the alloys.
4. The PEO film formed on Ca-containing specimens exhibited higher bioactivity, as indicated by the formation of a thicker apatite layer in SBF, because of the incorporation of Ca compounds into the film, as well as the presence of Ca in the alloys. Acceleration of apatite-layer growth was beneficial for decelerating the long-term corrosion rate in physiological solution.

## Figures and Tables

**Table 1:** Chemical composition (wt %) of rolled-plate AM60 magnesium alloy.

Mg	Al	Zn	Mn	Cu	Ni	Si	Be
Bal.	5.6	≤0.2	0.26	≤0.008	≤0.001	≤0.08	≤0.0005

**Table 2:** Ionic composition of SBF10 [16].

Ion	Na^+^	K^+^	Mg^+^	Ca^+^	Cl^−^	HCO_3_^−^	HPO_4_^2−^	SO_4_^2−^
Concentration (mM)	142	5	1	2.5	126	10	1	1
